# Identification and validation of key biomarkers associated with immune and oxidative stress for preeclampsia by WGCNA and machine learning

**DOI:** 10.3389/fgene.2025.1500061

**Published:** 2025-03-12

**Authors:** Tiantian Yu, Guiying Wang, Xia Xu, Jianying Yan

**Affiliations:** ^1^ College of Clinical Medicine for Obstetrics & Gynecology and Pediatrics, Fujian Medical University, Fujian Maternity and Child Health Hospital, Fuzhou, Fujian, China; ^2^ Fujian Clinical Research Center for Maternal - Fetal Medicine, Fuzhou, Fujian, China; ^3^ National Key Obstetric Clinical Specialty Construction Institution of China, Fuzhou, Fujian, China

**Keywords:** leptin, biomarker, preeclampsia, immune infiltration, machine learning, WGCNA

## Abstract

**Background:**

Preeclampsia (PE), a major obstetric disorder marked by dysfunction in both placental and maternal vascular systems, continues to pose critical challenges in global maternal healthcare. This multisystem pregnancy complication contributes significantly to adverse perinatal outcomes and remains a leading cause of pregnancy-related morbidity worldwide. However, the available treatment options at present remain restricted. Our investigation employs an integrative bioinformatics approach to elucidate critical molecular signatures linked to the interplay between immunological dysregulation and oxidative stress mechanisms in PE pathogenesis.

**Methods:**

In this study, we sourced the dataset from the GEO database with the aim of pinpointing differentially expressed genes (DEGs) between PE samples and control samples. Genes associated with oxidative stress were procured from the Genecards database. Next, we employed a comprehensive approach. This involved integrating WGCNA, GO and KEGG pathway analyses, constructing PPI networks, applying machine learning algorithms, performing gene GSEA, and conducting immune infiltration analysis to identify the key hub genes related to oxidative stress. Diagnostic potential of candidate biomarkers was quantitatively assessed through ROC curve modeling. Additionally, we constructed a miRNA - gene regulatory network for the identified diagnostic genes and predicted potential candidate drugs. In the final step, we validated the significant hub gene using independent external datasets, the hypoxia model of the HTR-8/SVneo cell line, and human placental tissue samples.

**Results:**

At last, leptin (LEP) was identified as a core gene through screening and was found to be upregulated. The results of quantitative real-time polymerase chain reaction (qRT -PCR) and immunohistochemistry validation were consistent with those obtained from the datasets. KEGG analysis revealed that LEP was significantly enriched in “allograft rejection,” “antigen processing,” “ECM receptor interaction” and “graft *versus* host disease.” GO analysis revealed that LEP was involved in biological processes such as “antigen processing and presentation,” “peptide antigen assembly with MHC protein complex,” “complex of collagen trimers,” “MHC class II protein complex” and “mitochondrial protein containing complex.” Moreover, immune cell analysis indicated that T follicular helper cells, plasmacytoid dendritic cells, neutrophils, and activated dendritic cells were positively correlated with LEP expression, whereas γδT cells, eosinophils, and central memory CD4^+^ T cells showed a negative correlation. These findings suggest that LEP influences the immune microenvironment of PE through its interaction with arious immune cells. In addition, 28 miRNAs and 15 drugs were predicted to target LEP. Finally, the overexpression of LEP was verified using independent external datasets, the hypoxia model of the HTR-8/SVneo cell line, and human placental tissue.

**Conclusion:**

Through an integrated analytical framework employing WGCNA coupled with three distinct machine learning-driven phenotypic classification models, we discovered a pivotal regulatory gene. This gene has the potential to act as a novel diagnostic biomarker for PE. Moreover, it can be considered as a promising target for drug development related to PE. Notably, it shows a strong correlation with the immune microenvironment, suggesting its crucial role in the complex pathophysiological processes underlying PE.

## 1 Introduction

Preeclampsia (PE) is a pregnancy-specific, multisystemic disorder characterized by clinical manifestations such as new-onset hypertension and proteinuria after 20 weeks of gestation. It stands as a significant contributor to maternal and fetal mortality rates (Mol et al., 2016). The incidence of PE varies based on geographic location, season, nutritional factors, and racial/ethnic backgrounds; however, it impacts approximately 3%–5% of women globally (Mol et al., 2016). Maternal complications associated with PE encompass eclampsia, renal failure, and hemolysis, elevated liver enzymes, and low platelet count (HELLP) syndrome. The pathophysiological mechanisms of PE encompass endothelial damage, systemic arteriolar spasm, reduced systemic perfusion, and multiorgan dysfunction, which collectively pose a significant threat to the health of both mothers and infants (San Juan-Reyes et al., 2020; Ziganshina et al., 2020).Currently, the only effective treatment is the termination of pregnancy (Tannetta and Sargent, 2013). Unfortunately, the exact etiology and pathogenesis remain unelucidated. However, placental dysfunction, characterized by inadequate trophoblast invasion of spiral arterioles, is thought to play a central role. This dysfunction may be triggered by an imbalance between the production and deactivation of reactive oxygen species (ROS) in the placenta (Mol et al., 2016; Roberts et al., 1989; Rana et al., 2019; Brown et al., 2018).

**FIGURE 1:**
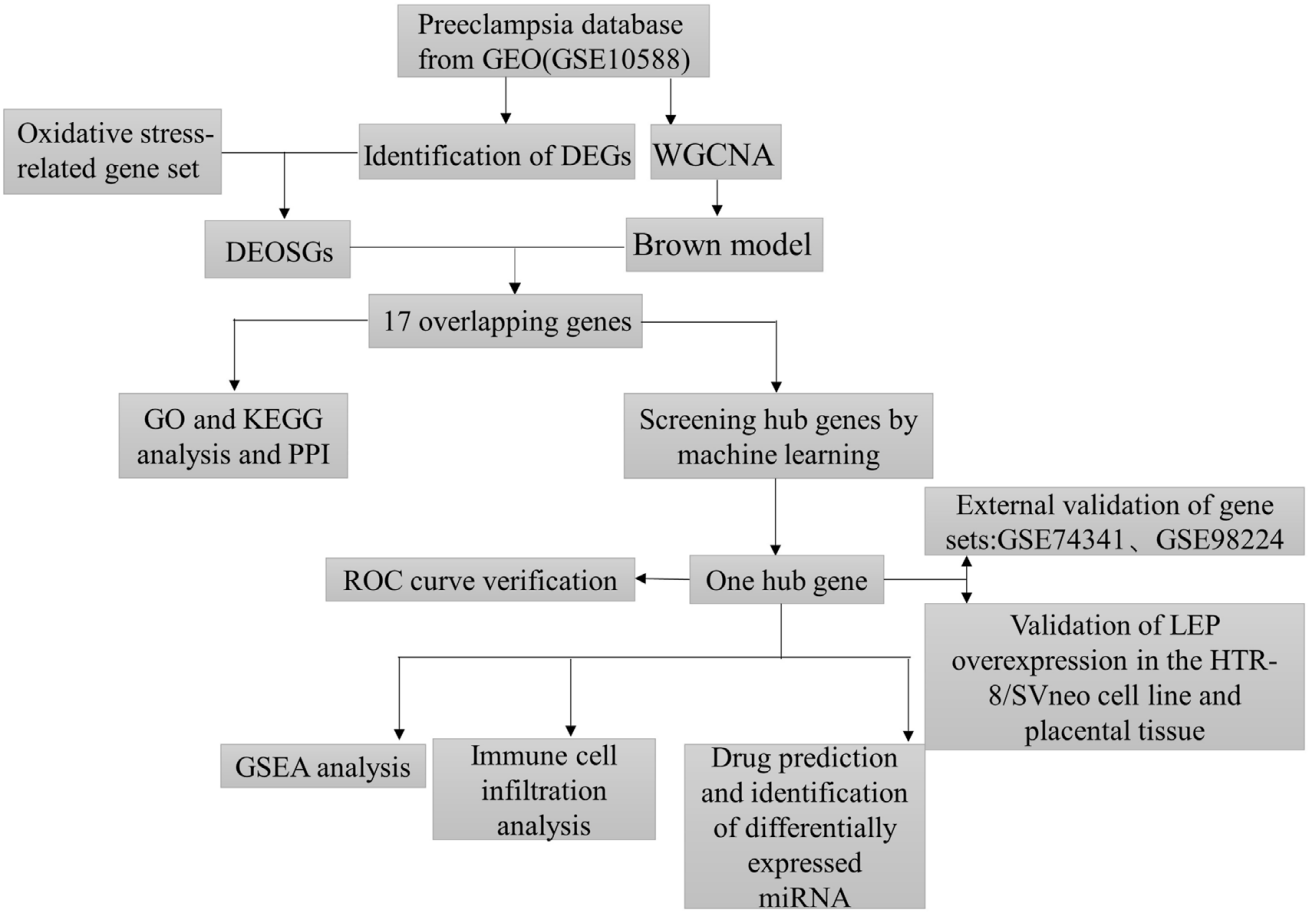
The flow chart of this study.

Oxidative stress plays a significant pathogenic role in PE, with numerous oxidative stress markers showing diagnostic potential (Agrawal et al., 2018). A substantial body of evidence underscores the crucial involvement of immune responses and oxidative stress in the development of PE (Meng et al., 2021; Afrose et al., 2022; Deer et al., 2021a). The integration of immune infiltration, oxidative stress, and bioinformatics approaches provides new insights into the diagnosis and treatment of PE. Furthermore, two external datasets were utilized to validate the identified gene. Ultimately, the gene was validated through quantitative real-time polymerase chain reaction (qRT -PCR) and immunohistochemistry. The findings of this study might offer new perspectives on the role of oxidative stress in placental pathology and could also facilitate the identification of potential biomarkers and therapeutic targets, providing a novel approach to the clinical diagnosis and treatment of PE. The study route is illustrated in Figure 1.

## 2 Materials and methods

Human PE datasets were extracted from the Gene Expression Omnibus (GEO) database (http://www.ncbi.nlm.nih.gov/geo/) in July 2023. Three PE datasets were retrieved: GSE10588 (GPL2986), consisting of 17 cases of PE and 26 control cases; GSE74341 (GPL16699), containing 12 PE cases and 10 control cases for validation; and another validation set GSE98224 (GPL6244)) containing 30 PE cases and 18 control samples. Furthermore, 1142 oxidative stress-related genes were extracted from the Genecards database. Pertinent details information is displayed in Table 1.

**TABLE 1:** Summary of the datasets included in this study and their features.

Dataset	Database	Platform	Sample
GSE10588	GEO	GPL2986	17 cases of PE and 26 controls
GSE74341	GEO	GPL16699	12 cases of PE and 10 controls
Oxidative stress-related genes	Genecard	Genecard	Obtaining oxidative stress-related genes from Genecard
GSE98224	GEO	GPL6244	30 cases of PE and 18 controls

### 2.1 Identification of differentially expressed genes (DEGs)

After normalizing the data, the “limma” R package (version 3.44.3) was used to perform differential expression analysis. The filter criteria were set at |Log2FC|>0.5 and padj<0.05 (Ritchie et al., 2015). The expression heat map of DEGs was generated by employing the “Pheatmap” R package (version 4.1.0) (Ritchie et al., 2015).

Subsequently, differentially expressed genes related to oxidative stress (DEOSGs) were identified by intersecting the DEGs with the 1142 oxidative stress-related genes.

### 2.2 WGCNA

Weighted gene co-expression network analysis (WGCNA) is an algorithm that can screen candidate biomarkers and the co-expressed gene modules with high correlation coefficients (Langfelder and Horvath, 2008). In this study, the “WGCNA” package (version 4.0.3) was used to identify key module and hub genes associated with PE using the GSE10588 dataset. To construct the network, the co-expression relationship was calculated by Pearson’s correlation coefficient. The optimal soft-thresholding factor (β) was chosen to strengthen strong correlations among the DEGs while penalizing the impact of weak correlations. The adjacency matrix was then converted into a Topological Overlap Matrix (TOM). Using the TOM-based dissimilarity measure, genes with similar expression patterns were grouped into distinct modules through hierarchical clustering. A module with a strong correlation to PE was identified based on gene significance and its correlation with clinical subtypes. The genes from this module were subsequently utilized for further analysis.

Eventually, the genes from DEOSGs and key modules were intersected.

### 2.3 Construction of the PPI network and functional enrichment analysis

Gene Ontology (GO) analyse serves as a comprehensive repository of computable knowledge concerning the functions of genes and gene products (The Gene Ontology Consortium, 2019). Kyoto Encyclopedia of Genes and Genomes (KEGG) analyse is a widely utilized database for pathway enrichment analysis (Kanehisa and Goto, 2000). GO and KEGG analyses were executed using Metascape (http://metascape.org).

In addition, the GeneMANIA (http://genemania.org/search/) online database was utilized to construct a gene interaction network and analyze the functions of the identified genes. The protein-protein interaction networks (PPI) network was built to examine the interactions between protein-coding genes using the GeneMANIA tool.

### 2.4 Screening hub genes by machine learning and ROC curve analysis

To identify key biomarkers, we employed several machine learning methods. Least Absolute Shrinkage and Selection Operator (LASSO) logistic regression analysis is a data mining technique that uses an L1 penalty to eliminate less important variables. This approach helps in selecting significant variables for classification models (Zhang et al., 2021). Support Vector Machine-Recursive Feature Elimination (SVM-RFE) analysis is a supervised machine-learning approach that progressively removes less informative features to identify the most crucial genes (Lin et al., 2017). Random Forest (RF) is a suitable methodology that operates without constraints on variable conditions and evaluates the importance of each gene by assessing how well it contributes to the model’s predictive accuracy across multiple decision trees. It is another machine learning method that works well for prediction tasks with continuous variables, offering high accuracy, sensitivity, and specificity (Ellis et al., 2014). These algorithms complement each other by offering different perspectives on feature selection. While LASSO provides penalization to eliminate irrelevant features, Random Forest evaluates feature importance in terms of model accuracy, and SVM-RFE systematically eliminates less relevant features. By using these three methods in parallel, we aimed to increase the robustness of the selected biomarkers and ensure that the identified biomarkers are consistently relevant across different algorithmic approaches. LASSO regression and RF analysis were conducted using the “glmnet” (Zhang et al., 2019) (version 3.3.3) and “randomForest” (Alderden et al., 2018) (version 3.0.2) R packages. The intersection of the key genes identified by the three algorithms was used for further analysis.

Furthermore, receiver operating characteristic (ROC) curves and the area under the curve (AUC) were employed to assess the diagnostic efficacy. Ultimately, the gene was recognized as a hub gene through both methods combined.

### 2.5 GSEA of biological functions and pathways of diagnostic markers

Gene Set Enrichment Analysis (GSEA) is a computational method to analyze gene sets and identify biological functions and signaling pathways (Subramanian et al., 2005). Samples were categorized into high- and low-expression groups based on the gene expression of each diagnostic marker. A GSEA was then conducted to investigate the related biological functions and pathways, with *P* < 0.05 set as the significance threshold.

### 2.6 Assessment of the immune landscape

To quantify the immune response, we used single-sample GSEA (ssGSEA) to analyze 28 immune-related gene sets. The gene set encompassing 28 immune cell types was derived from a previously published article (Charoentong et al., 2017). Using ssGSEA, a comparison was performed on differential immune cell infiltration patterns and evaluate functional variations in immunoregulatory pathways between cohorts dichotomized by transcript abundance thresholds. The “GSVA” R package was used for this analysis, with *P* < 0.05 indicating statistical significance.

### 2.7 Prediction of potential drugs and construction of diagnostic gene-miRNA network

We utilized the DGIdb (https://www.dgidb.org), a Drug-Gene interaction database (Cotto et al., 2018), to identify potentially druggable targets based on the biomarkers for PE. The gene-drug network was visualized using Cytoscape software (version 3.9.0).

To computationally identify miRNA-mRNA interactions of diagnostic significance, we performed multi-platform target prediction analyses integrating miRanda (http://www.microrna.org/), miRDB (http://mirdb.org/),and TargetScan (http://www.targetscan.org/vert_72/). Consensus predictions derived from tripartite database intersection were subsequently modeled as regulatory networks using Cytoscape (v3.9.0), a graph theory-based visualization platform for biomolecular interaction mapping.

### 2.8 Validation of independent external datasets

To validate the findings, the expression of key hub genes was verified in external datasets, GSE74341 and GSE98224.

### 2.9 qRT -PCR assays for gene expression in cell lines

The HTR8/SVneo (CRL-3271; ATCC) cell line was cultured in RPMI-1640 medium (Glibco) supplemented with 5% fetal bovine serum (Glibco) and maintained with antibiotics (100 U/mL penicillin and 100 μg/mL streptomycin). Cells were then incubated at 37°C in 5% CO_2_ for a duration of 24 h. For experiments involving hypoxia, cells were cultured under serum-free conditions at 37°C in a humidified 3-gas incubator containing 1% O_2_ and 5% CO_2_. This experimental condition was selected based on prior research (Koklanaris et al., 2006; Graham et al., 1998), and was aimed at simulating the conditions of PE for a period of 24 h.

Total RNA was extracted utilizing TRIzol reagent (Thermo Fisher Scientific). Complementary DNA (cDNA) was synthesized through reverse transcription using Prime-Script RTase (Takara). Gene expression was normalized to actin and analyzed using the 2^−ΔΔCt^ method. Primer sequences employed for qRT -PCR amplification are mentioned below:

ACTIN
  Forward: 5′- TCC​GCC​CCG​CGA​GCA​CAG​AG-3′
  Reverse: 5′- TCA​TCA​TCC​ATG​GTG​AGC​TGG​CGG​C -3′,
LEP
  Forward: 5′- TCC​TCA​CCA​GTA​TGC​CTT​CC -3′
  Reverse: 5′- TCT​GTG​GAG​TAG​CCT​GAA​GG-3′

### 2.10 IHC

The immunohistochemical (IHC) analysis in this study was conducted using placental samples from four PE patients and four healthy controls from Fujian Provincial Maternity and Child Healthcare Hospital to validate our bioinformatics results. The study was reviewed and approved by the Ethics Committee of Fujian Provincial Maternity and Child Healthcare Hospital (2024KY070-02). Fresh placental samples were immediately fixed in 4% paraformaldehyde (PFA, Sigma-Aldrich) at 4°C for 24 h and subsequently embedded in paraffin matrix. Thin sections (4 μm) mounted on charged slides underwent standard deparaffinization procedures followed by antigen unmasking via microwave-assisted retrieval in EDTA buffer (pH 8.0). After hydrogen peroxide-mediated peroxidase inactivation and 10% normal goat serum blocking, sections were probed with leptin-specific rabbit polyclonal antibody (ABclonal, 1:200 dilution) through 4°C overnight incubation. Subsequently, the sections were incubated with the secondary antibody at room temperature for 1.5 h, followed by incubation with 3,3′-diaminobenzidine at room temperature for 20 min. The stained sections were visualized using an optical microscope (Leica), and quantitative analysis of the samples was carried out with the assistance of ImageJ software (version 1.52).

## 3 Results

### 3.1 DEGs in the placenta samples

**FIGURE 2:**
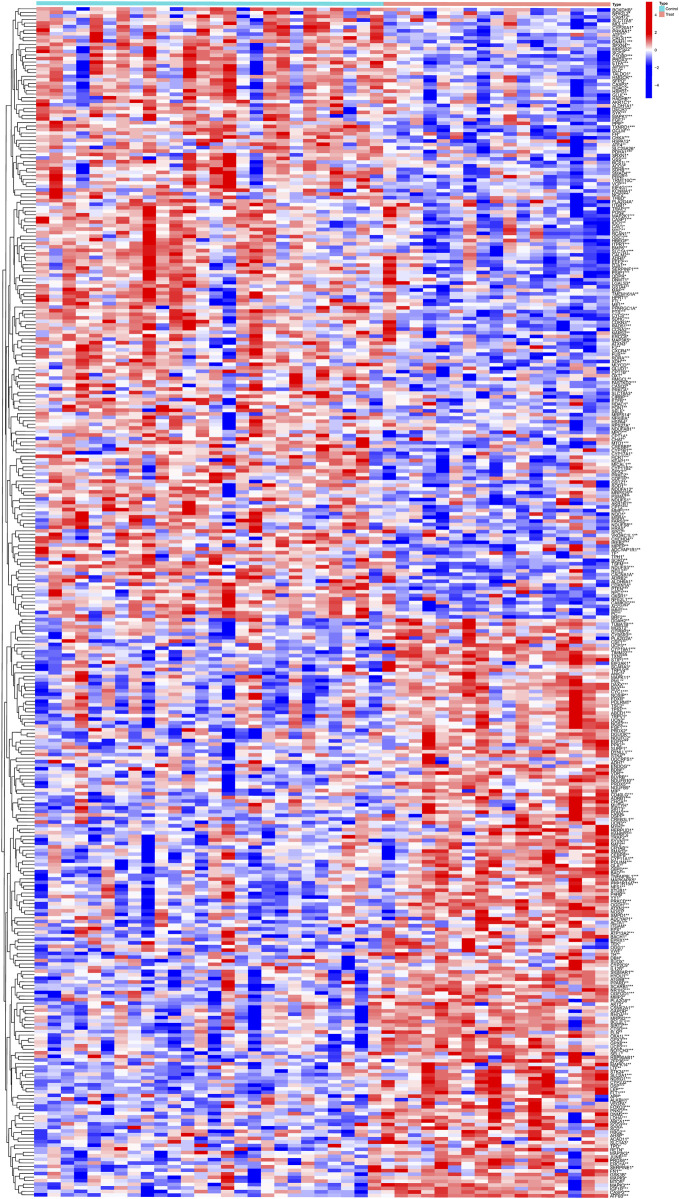
Clustered heatmap of PE-related DEGs expression levels.

Gene expression data in the placental tissues of individuals with non-PE and PE were retrieved from the GSE10588 dataset in the GEO databases. The integrated dataset consists of 17 PE samples and 26 control samples. A total of 335 DEGs were identified utilizing the Limma method. The heatmap of DEGs is shown in Figure 2.

### 3.2 Identification of co-expression modules utilizing WGCNA

**FIGURE 3:**
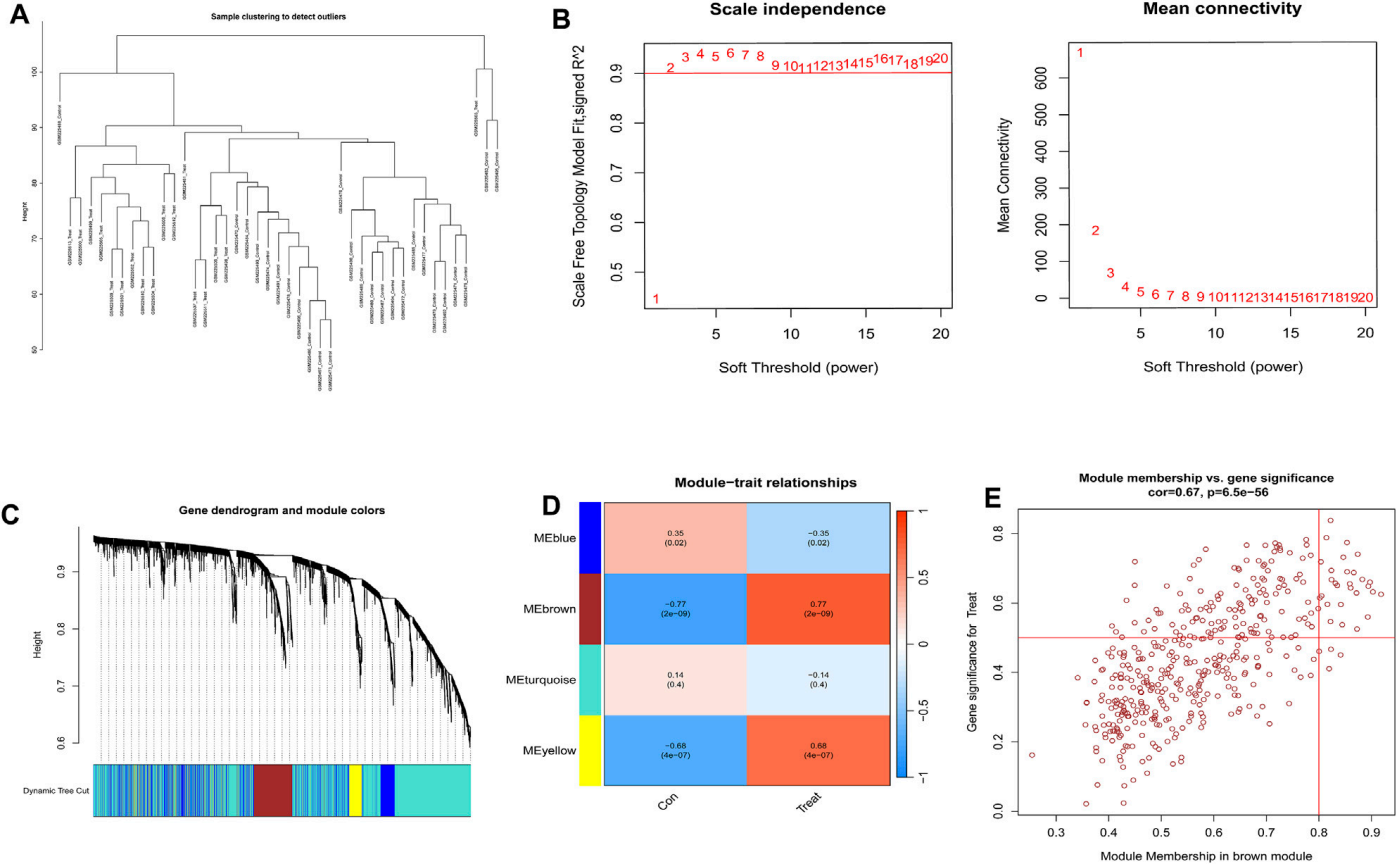
Construction of weighted gene co-expression networks. **(A)** Sample clustering and detection of outliers. **(B)** In the process of choosing the soft-threshold power, two aspects were analyzed: the scale-free fit index (displayed on the left) and the mean connectivity (presented on the right). **(C)** Clustering of co-expression modules was conducted. Each branch in the graph corresponds to a single gene. Genes grouped into the identical module were designated with the same color. **(D)** Correlations between the modules and normal placenta as well as preeclampsia placenta. **(E)** The relevance of members in the brown module and preeclampsia.

The gene expression profiles of the PE samples obtained from the GSE10588 dataset were analyzed using the WGCNA technique. This analysis involved constructing a gene co-expression network and identifying co-expression network and identifying co-expression modules using the WGCNA package in R. The dataset comprising 26 normal samples and 17 PE samples, which underwent clustering. Samples exhibiting evident aberrations were excluded based on a predefined threshold, as depicted in Figure 3A. Furthermore, a soft threshold of β = 4 (scale-free R^2^ = 0.9) was employed to construct a scale-free network, ensuring high connectivity (Figure 3B). Next, four modules were developed based on a gene clustering tree and a dynamic hybrid cutting algorithm (with a minimum of 100 genes per module) (Figure 3C). The module with the highest correlation with PE was brown, r = 0.77, *P* = 2e-09 (Figure 3D). The significance of the genes in the brown module with the PE genes was cor = 0.67, *P* = 6.5e-56 (Figure 3E).

### 3.3 Functional enrichment analysis and PPI network

**FIGURE 4:**
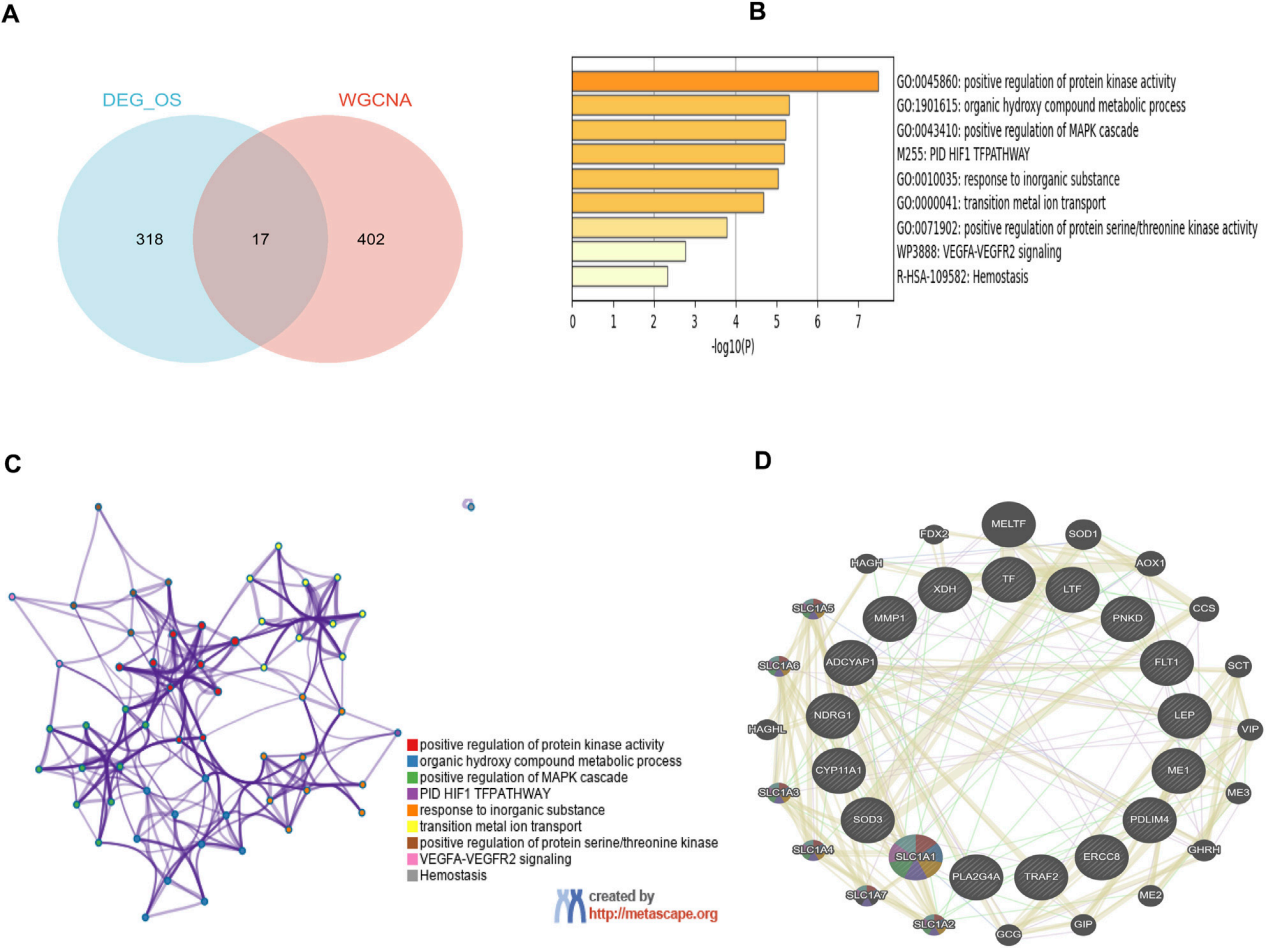
Functional enrichment analysis of key module genes merged with DEOSGs. **(A)** Venn diagram of key module genes *versus* DEOSGs. **(B)** Bar plot of DEOSGs functional enrichment terms. **(C)** Network relationship plots among all terms. **(D)** The PPI network of 17 genes constructed by GeneMANIA. The 20 most frequently changed neighboring genes are shown.

17 overlapping genes were identified by intersecting the critical module genes and DEOSGs utilizing a Venn diagram (Figure 4A). GO and pathway analyses were then performed to explore the biological functions of these overlapping genes using Metascape. The overlapping genes were primarily involved with positive regulation of protein kinase activity, positive regulation of MAPK cascade, organic hydroxy compound metabolic process, response to inorganic substance, transition metal ion transport, and various other biological processes (Figure 4B). Figure 4C revealed the relationships between the GO terms. The PPI network visually depicted the interactions among these 17 hub genes that involved in the development of PE (Figure 4D).

### 3.4 Selection of feature genes and GSEA analysis of potential biomarkers

**FIGURE 5:**
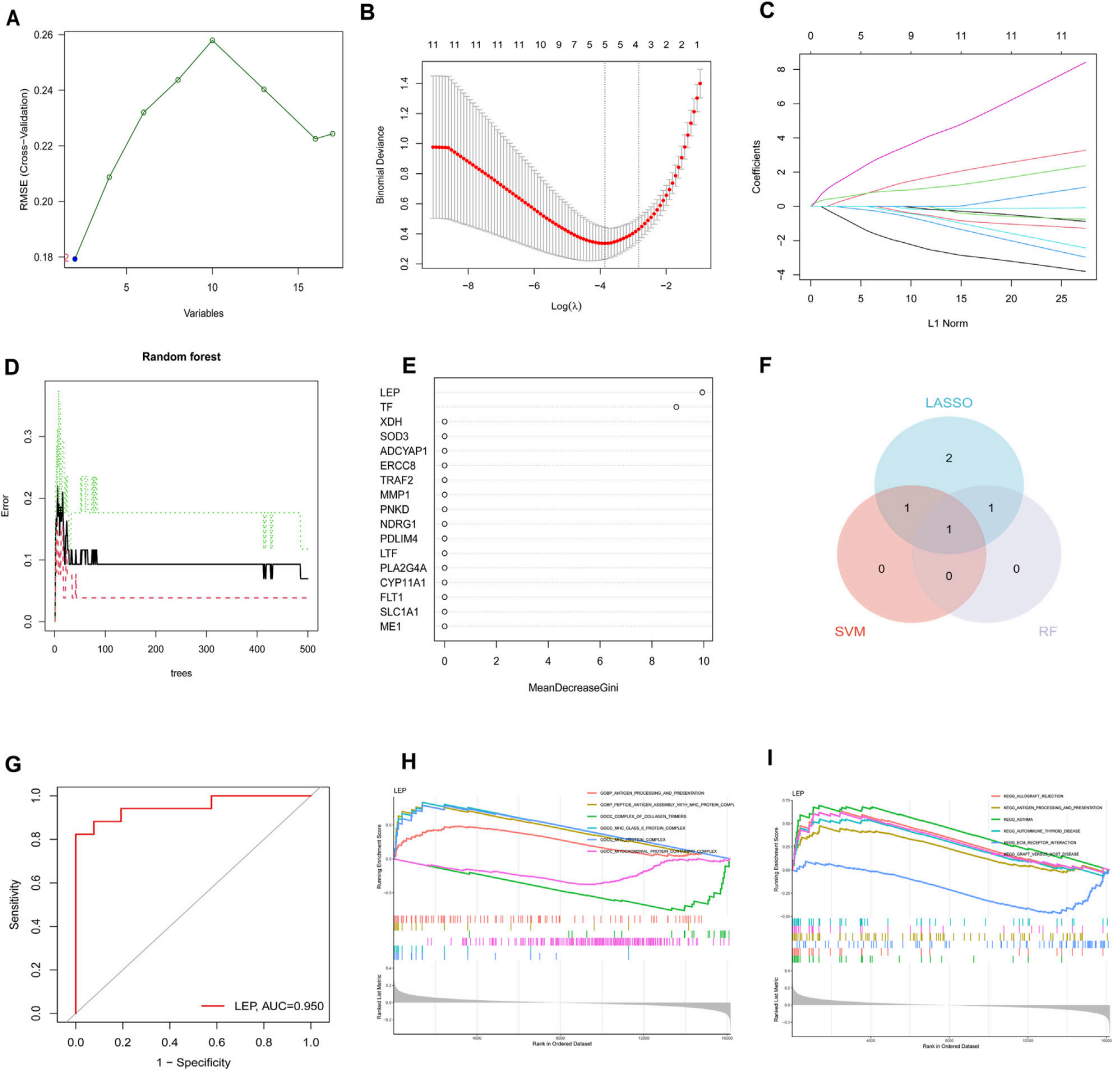
Screening hub genes by machine learning and GSEA analysis of potential biomarkers. **(A)** SVM-RFE algorithm. **(B, C)** LASSO regression algorithm. **(D, E)** RF algorithm. **(F)** Venn diagrams for three algorithms. **(G)** Hub gene in the GSE10588 dataset were analyzed using ROC curves. **(H)** GO analysis results for LEP. **(I)** KEGG analysis results for LEP.

In this study, three machine-learning algorithms were utilized to identify feature genes: SVM-RFE (Figure 5A); LASSO regression analysis, which selected five genes based on statistically significant univariable analyses (Figures 5B, C); and the RF algorithm, which ranked genes based on their importance (Figures 5D, E). The Venn diagram displayed the overlap of the three approaches, resulting in the identification of a single gene, namely leptin (LEP)(Figure 5F). Subsequently, a logistic regression model was developed using this candidate gene. The outcomes showcased the superior diagnostic performance of the predictive model, with an AUC of 0.95 in the training set (Figure 5G). These findings suggest that LEP may serve as a promising biomarker for PE.

Signaling pathways related to the characteristic genes were analyzed using GSEA. KEGG analysis revealed that LEP was significantly enriched in pathways such as “allograft rejection,” “antigen processing,” “asthma,” “autoimmune thyroid disease,” “ECM receptor interaction,” and “graft *versus* host disease” (Figure 5H). GO analysis identified key biological processes including “antigen processing and presentation,” “peptide antigen assembly with MHC protein complex,” “complex of collagen trimers,” “MHC class II protein complex,” “MHC protein complex,” and “mitochondrial protein-containing complex” (Figure 5I).

### 3.5 Evaluation and analysis of immune cell infiltration using ssGSEA and potential drug prediction

**FIGURE 6:**
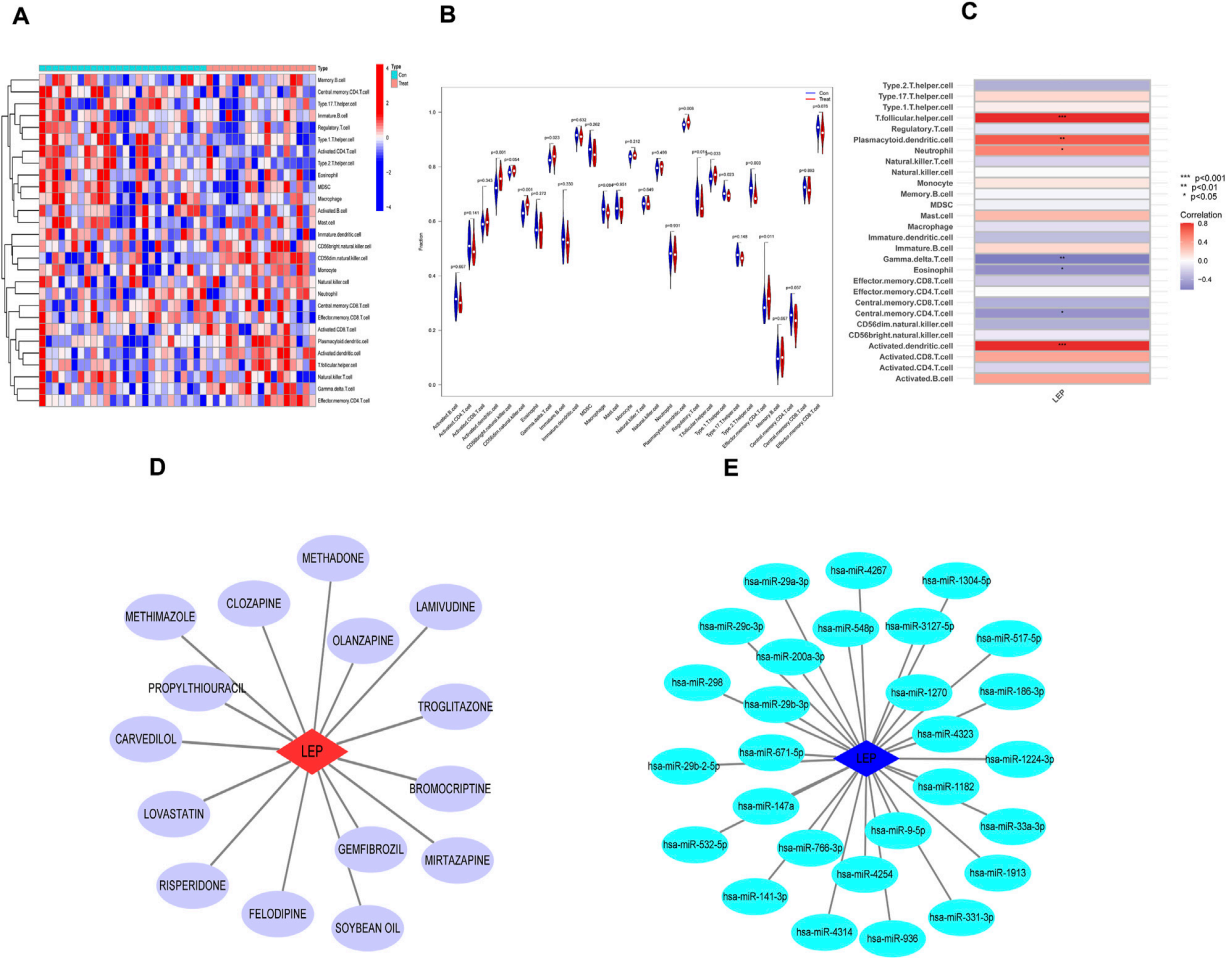
Immune infiltration analysis and potential drug prediction. **(A)** Heatmap showed the composition of 28 kinds of immune cells in each sample. **(B)** Comparison regarding the proportion of 28 kinds of immune cells between PE and normal groups visualized by the vioplot. **(C)** Heatmap of the correlations between the biomarkers and infiltrating immune cells. **(D)** Hub gene–potential drug network; the red node represents the hub gene, and the violet node represents the potential drug. **(E)** Interaction between candidate genes and miRNA (*, *p* < 0.05; **, *p* < 0.01; ***, *p* < 0.001).

To further investigate immune infiltration differences between individuals with PE and healthy controls, we performed ssGSEA. Figure 6A illustrates the distribution of 28 immune cell types in the GSE10588 dataset. Notably, PE samples exhibited significantly higher infiltration levels of several immune cell types, including activated dendritic cells, CD56^dim^ natural killer cells, γδT cell, regulatory T cells, T follicular helper cells, type 1 T helper cells, type 2 T helper cells, and effector memory CD4^+^ T cells, compared to healthy controls. This suggests that these immune cell types play a central role in PE development (Figure 6B). Furthermore, a positive correlation was observed between LEP expression and T follicular helper cells, plasmacytoid dendritic cells, neutrophils, and activated dendritic cells, while γδT cells, eosinophils, and central memory CD4^+^ T cells exhibited a negative correlation with LEP (Figure 6C).

To identify potential therapeutic drugs for PE, we searched the DGIdb database and identified 15 potential drugs that target LEP. Cytoscape was employed to visualize the drug-gene interaction networks for enhanced clarity and comprehensibility (Figure 6D). However, the underlying mechanism linking these potential drugs to LEP remain unclear. Subsequently, a search of the miRDB, miRanda, and TargetScan databases identified 28 miRNAs that may target LEP. The networks depicting these interactions were visualized using Cytoscape (Figure 6E).

### 3.6 Validation of independent external datasets and experimental

**FIGURE 7:**
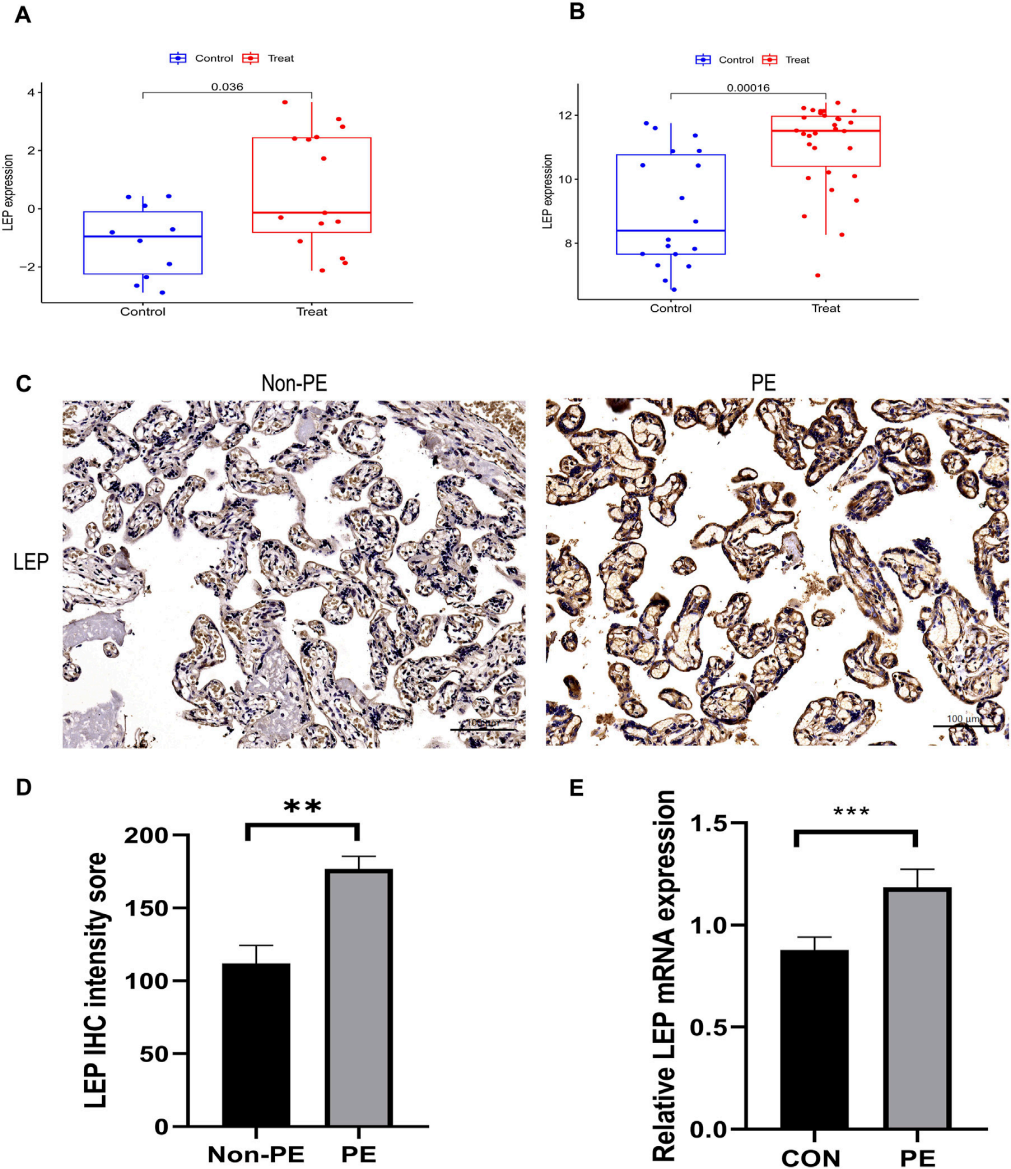
Validation of hub gene. **(A)** The hub gene in GSE74341 showed significant differences, with *p* value <0.05. **(B)** The hub gene in GSE98224 showed significant differences, with *p* value <0.05. **(C)** Immunohistochemical staining of placental tissue. **(D)** Histograms of immunohistochemical staining results. **(E)** Validation of LEP expression in the HTR-8/SVneo cell line by qRT-PCR analysis of LEP expression under normal oxygen concentration and hypoxia, CON: normal oxygen concentration for 24 h, hypoxia: hypoxia condition for 24 h (**, *p* < 0.01; ***, *p* < 0.001). Scale bars = 100 μm.

Two independent external datasets (GSE74341 and GSE98224) were utilized to validate the analytical results. The findings revealed that LEP expression was significantly upregulated in placental tissues of PE patients (Figures 7A, B).

To validate the bioinformatics results, immunohistochemistry was used to assess the levels of LEP protein in preeclamptic placenta samples (Figure 7C). The results indicated that LEP protein expression was upregulated in preeclamptic placenta samples compared to the control group (Figure 7D).

To further explore the expression of LEP in PE, a hypoxia model was established utilizing the HTR-8/SVneo cell line. Subsequently, LEP expression within this model was evaluated using qRT-PCR. The findings demonstrated a significant upregulation of LEP mRNA expression under hypoxic conditions (Figure 7E).

## 4 Discussion

PE represents a multifaceted clinical syndrome that is exclusive to human pregnancy and plays a major role in causing morbidity and mortality among pregnant women and their neonates (Roberts et al., 1989). Despite its significance, the exact pathogenesis of PE remains incompletely understood, resulting from intricate interactions of various factors. PE can be triggered by a number of conditions such as systemic inflammation, placental dysfunction, hypoxia, immunological dysregulation, and oxidative stress (Jung et al., 2022; George and Granger, 2011).Oxidative stress molecular markers play a critical role in the pathogenesis of PE. During early pregnancy, abnormal invasion of trophoblasts into the maternal uterine spiral arteries leads to oxidative stress, enhancing the production of oxygen-free radicals in the placental environment (Karaşin and Çift, 2020; van Rijn et al., 2008). Certain studies have also revealed that immune response disruptions have a remarkable impact on the development of this obstetrical syndrome (Miller et al., 2022; Santana-Garrido et al., 2022; Lang et al., 2022; Deer et al., 2021b). Furthermore, advances in informatics technologies now allow the identification of diagnostic markers associated with both immune responses and oxidative stress in the context of PE.

WGCNA is a widely used method for bioinformatics analysis. In this study, We WGCNA algorithm to build a gene co-expression network, subsequently leveraging hierarchical clustering for delineating functional modules enriched with tightly interconnected genes. Analysis of the WGCNA results revealed that genes in the brown module exhibited the highest correlation with PE development. After that, a Venn diagram was used to disclose 17 genes that overlapped among the gene sets under study. GO functional analysis illustrated that these genes were involved in processes such as the positive regulation of organic hydroxy compound metabolic process, protein kinase activity, and MAPK cascade positive regulation. Similarly, KEGG analysis demonstrated that these genes were primarily associated with the positive regulation of protein kinase activity, organic hydroxy compound metabolism, positive regulation of MAPK cascade, response to inorganic substance, transition metal ion transport, and several other biological processes. Subsequently, machine-learning techniques, including LASSO, RF, and SVM-RFE, were employed to identify key disease-related genes. Furthermore, ROC curves and AUC values were utilized to assess diagnostic potential of these markers. Ultimately, a potential diagnostic marker for PE, namely, LEP, was identified.

Currently, identifying effective early prediction methods for PE remains a significant challenge. A cohort study conducted in the United States found that the maternal LEP/ceramide (cer) ratio in early pregnancy serves as a superior non-invasive serum biomarker for predicting PE, compared to the sFlt/PlGF ratio (Huang et al., 2021). This suggests that LEP may be more helpful than sFlt/PlGF in predicting PE.LEP is a protein-coding gene released by white adipocytes into the bloodstream, where it plays a crucial role in maintaining energy balance.LEP possesses various endocrine functions and isimplicated in the regulation of immune and inflammatoryresponses, hematopoiesis, angiogenesis, reproduction, anthe promotion of angiogenesis (Pérez-Pérez et al., 2018; Fasshauer and Blüher, 2015; Nieuwenhuis et al., 2020; Childs et al., 2021)^.^LEP acts on variety of immune cells, influencing their development and function, and plays a key role as an immune modulator in the body. Most immune cells express LEP receptors and respond to LEP. In particular, LEP is essential for the development, function, and metabolism of T cells. Research indicates that LEP is critical for the early development of T cells, but is not required for CD8^+^ T cells (Kim et al., 2010). It has been demonstrated that leptin receptor signaling in T cells enhances cell survival, differentiation, cytokine production, and proliferation differentiatio (Fujita et al., 2002; Saucillo et al., 2014; Lord et al., 1998; Gerriets et al., 2016). Furthermore, LEP promotes B cell homeostasis by inhibiting apoptosis and promoting cell cycle progression (Lam et al., 2010). *In vitro* studies have demonstrated that LEP can activate B cells, inducing a pro-inflammatory phenotype (Frasca et al., 2020; Agrawal et al., 2011).LEP also binds specifically to LEP receptors on macrophages, promoting their phagocytic activity (Mancuso et al., 2018). Similar to T cells, LEP activation in monocytes induces the expression of an inflammatory phenotype (Jaedicke et al., 2013). LEP has been shown to promote the anti-apoptotic effects of dendritic cells and enhance their maturation (Mattioli et al., 2005; Moraes-Vieira et al., 2014). Studies also indicate that LEP can inhibit neutrophil apoptosis and act as a chemoattractant for neutrophils (Bruno et al., 2005; Ubags et al., 2014; Naylor et al., 2014). In addition, LEP attracts eosinophils and basophils in a manner similar to its effect on neutrophils (Suzukawa et al., 2011; Kato et al., 2011). Furthermore, LEP plays a critical role in the development of natural killer (NK) cells (Tian et al., 2002). Currently, no studies have reported LEP-induced oxidative stress activation in existing PE animal models. However, LEP has been shown to increase ROS levels in vascular smooth muscle cells and promote cell proliferation through the activating of thethe NF-κB pathway (Schroeter et al., 2012; Li et al., 2005). In the placenta of PE patients, an increase in ROS levels can be observed, potentially due to the activation of the NF-κB pathway in trophoblast cells, leading to enhanced ROS production (Wang et al., 2021). Excessively high LEP levels may disrupt glucose and lipid metabolism, leading to the excessive release of lipid peroxides and ROS, which increases the risk of PE (Kutlu et al., 2005). This may help explain our findings. The inflammatory microenvironment in PE patients promotes the infiltration of dendritic cells. However, the increased proportion of conventional dendritic cells and their over-maturation in PE patients may contribute to the increased production of pro-inflammatory cytokines and maternal damage (Zhang et al., 2017; Darmochwal-Kolarz et al., 2003). This finding aligns with our results. Decidual NK (dNK) cells actively mediate two pivotal processes in early gestation: facilitating trophoblast migration and orchestrating structural transformation of spiral arteries, potentially contributing to the development of PE (Kalkunte et al., 2009).This process may be driven by the secretion of vascular endothelial growth factor (VEGF) and placental growth factor placental growth factor (PlGF) by dNK cells, which stimulate spiral artery remodeling (Kalkunte et al., 2009; Gibson et al., 2015). Our results indicate a higher proportion of CD56^dim^ NK cells in preeclamptic women, whereas no significant difference was observed in the proportion of CD56^bright^ NK cells between the PE and control groups. T cells are classified into αβ T cells and γδ T cells based on differences in their T cell receptors. Currently, the role of γδ T cells in the pathogenesis of PE remains unclear.; however, animal studies have shown a significant elevation in γδ T cells in the placenta of PE model mice, with γδ T cell-deficient mice exhibiting improved PE symptoms (Chatterjee et al., 2017). This finding is consistent with our results. During pregnancy, type 1 T helper cell (Th1) immunity exerts pro-inflammatory effects, while type 2 T helper cell (Th2) immunity has anti-inflammatory effects. The balance between Th1 and Th2 immune responses is crucial for a healthy pregnancy. However, in PE patients, the Th1/Th2 ratio in peripheral blood is increased (El-Kabarity and Naguib, 2011). A deficiency of decidual regulatory T cells (Treg cells) is associated with poor spiral artery remodeling and insufficient trophoblast invasion, both of which exacerbate the symptoms of PE (Harmon et al., 2016).

LEP plays a critical role in placental development and function, influencing processes such as invasion, protein synthesis, cell proliferation, and apoptosis in placental cells (Pérez-Pérez et al., 2018; D'Ippolito et al., 2012; Schanton et al., 2018). Multiple studies have underscored LEP’s involvement in the pathophysiology of PE (Pérez-Pérez et al., 2018; Dos Santos et al., 2015; Hauguel-de Mouzon et al., 2006; Gutaj et al., 2020). Notably, PE patients exhibit elevated serum LEP levels compared to individuals without the condition, and elevated levels of this protein have also been reported in the placentas of PE patients. (Song et al., 2016; Kalinderis et al., 2015; Taylor et al., 2015). Multiple investigations have detected both LEP mRNA and protein expression in the placentas of PE patients (Taylor et al., 2015; Mise et al., 1998; Nishizawa et al., 2007), findings consistent with the results of the present study.

On the one hand, the elevated LEP levels may reflect a compensatory response to placental underperfusion, aiming to support neovascularization (Eleuterio et al., 2014; Lepercq et al., 2003). On the other hand, LEP is known to play a role in immune regulation, and disruptions in immune homeostasis may lead to alterations in maternal LEP expression in PE. However, further comprehensive research is needed to fully elucidate the dysregulation of LEP in the context of PE and its broader impact on maternal physiology.

PE is a complex systemic condition, with increasing evidence highlighting the immune system’s pivotal role in its development (Lu and Hu, 2019). Thus, to further investigate this, an analysis of immune infiltration was conducted to compare the immune cell profiles between normal and PE samples. The results revealed a remarkable increase in the infiltration of eight immune cell types in PE samples compared to normal samples. These immune cell types included activated dendritic cells, type 1 T helper cells,CD56^dim^ natural killer cells, γδT cells, macrophages, regulatory T cells, T follicular helper cells, type 2 T helper cells, and effector memory CD4^+^ T cells.

These findings indicate that LEP may serve as a promising diagnostic and therapeutic marker for PE, potentially exerting a significant role in its pathogenesis. Nevertheless, the study lacks longitudinal data, which could be valuable in understanding the temporal changes in LEP expression and its correlation with disease severity and outcomes; validating the prognostic and therapeutic implications of this model will require a larger sample size, longitudinal data, and comprehensive clinical data from multiple medical center in future research. The potential therapeutic drugs targeting LEP in PE remains to be confirmed, and future research should focus on conducting fundamental studies or clinical trials to validate the feasibility of LEP-targeting drugs in the treatment of PE. Additionally, as this study relies on publicly available data, further experimental investigations are needed to elucidate the biological functions of LEP.

## 5 Conclusion

In summary, through integrated bioinformatics analyses and experimental verification, we identified LEP as a key biomarker associated with immune infiltration and oxidative stress in PE.

## Data Availability

The data presented in the study can be found in the Gene Expression Omnibus (GEO) datasets (http://www.ncbi.nlm.nih.gov/geo/), accession number GSE10588 (GPL2986), GSE74341 (GPL16699) and GSE98224 (GPL6244). Since we utilized existing public data rather than generating new data, there is no need to deposit data in a separate repository. We believe the information provided in the manuscript about the data sources is sufficient, and we are ready to move forward with the publication process.
